# Determinants of long‐term disability in chronic inflammatory demyelinating polyradiculoneuropathy: A multicenter Korea/UK study of 144 patients

**DOI:** 10.1111/ene.16575

**Published:** 2024-12-09

**Authors:** Young Gi Min, Jaehyun Jeon, Sung‐Min Kim, Yoon‐Ho Hong, Christina Englezou, Jung‐Joon Sung, Yusuf A. Rajabally

**Affiliations:** ^1^ Department of Translational Medicine Seoul National University College of Medicine Seoul Republic of Korea; ^2^ Department of Neurology Seoul National University Hospital Seoul Republic of Korea; ^3^ Department of Neurology Boramae Medical Center Seoul Republic of Korea; ^4^ Department of Neurology, Inflammatory Neuropathy Clinic University Hospitals Birmingham Birmingham UK; ^5^ Biomedical Research Institute Seoul National University Hospital Seoul Republic of Korea; ^6^ Neuroscience Research Institute Seoul National University College of Medicine Seoul Republic of Korea; ^7^ Wide River Institute of Immunology Seoul National University Hongcheon Gangwon‐do South Korea; ^8^ Aston Medical School Aston University Birmingham UK

**Keywords:** CIDP, clinical management, prognosis, treatment timing

## Abstract

**Background:**

Despite standard‐of‐care treatment, therapeutic outcomes in chronic inflammatory demyelinating polyradiculoneuropathy (CIDP) are often incomplete. We aimed to evaluate the impact of clinical and therapeutic factors on long‐term disability in CIDP, from cohorts from Korea and the UK.

**Methods:**

We conducted a retrospective multicenter cohort study of 144 patients with CIDP. Baseline characteristics and treatment data were collected, and disability was assessed using the Overall Neuropathy Limitation Scale (ONLS) for the UK cohort, Inflammatory Neuropathy Cause and Treatment (INCAT) scores for the Korean cohort, and Inflammatory Rasch‐built Overall Disability Scale (I‐RODS) for the combined cohort. Univariate and multivariate linear regression analyses were performed to identify independent prognostic factors. Subgroup analyses were conducted according to important clinical features to gain further insights into which patients are most likely to benefit from early treatment.

**Results:**

Treatment initiation within 1 year of onset was significantly associated with lesser post‐treatment disability and greater amplitude of treatment response, in each cohort separately, and in the combined cohort. This association remained significant after adjusting for covariates in multivariate regression. Subgroup analyses demonstrated early treatment benefits in older patients (≥60 years), those with typical CIDP, and those with a chronic mode of onset. The type of first‐line therapy and baseline disability levels did not influence outcomes. Other identified independent prognostic factors included comorbidity and pre‐treatment disability level.

**Discussion:**

Early treatment initiation is a key modifiable determinant of favorable long‐term disability in CIDP. These findings underscore the importance of timely diagnosis and prompt treatment to prevent irreversible axonal damage.

## INTRODUCTION

Chronic inflammatory demyelinating polyradiculoneuropathy (CIDP) is an immune‐mediated disorder characterized by muscle weakness and sensory impairment due to demyelination of peripheral nerves. First‐line treatments include intravenous or subcutaneous immunoglobulin (IV/SCIG), corticosteroids, or plasma exchange (PLEX). Despite standard care, therapeutic outcomes in CIDP are often incomplete. About 20–30% of patients do not respond to treatment, and over 60% continue to experience persistent disability. [1, 2, 3] Nearly 10% of patients remain unable to walk. [4] Given the clinical heterogeneity of CIDP, it is essential to understand the factors associated with outcomes and address those that are modifiable.

Various prognostic factors have been proposed, including pre‐treatment disability level, response to initial treatment, CIDP phenotype, and the acuteness of presentation. [5, 6, 7, 8, 9, 10, 11] A recent German study, along with earlier research, found that a shorter disease duration at the time of the first treatment could positively influence outcomes. [5, 9, 12] However, the implications of these studies were limited by the use of insensitive outcome measures, for example, the modified Rankin scale (mRS), small sample sizes, single‐center studies, and lack of consideration of potential covariates.

Using our cohorts from Korea and the UK, and applying multiple validated outcome measures, our primary goal was to evaluate the impact of clinical and therapeutic factors on outcomes in CIDP.

## METHODS

### Participants and data collection

We included treatment‐naïve patients who were diagnosed with CIDP according to the 2021 EAN/PNS guidelines (excluding “possible CIDP”) and who began treatment between June 2014 through August 2023 (Supplementary Figure 1) at three neuromuscular centers, in Seoul (Korea) and Birmingham (UK). [13] To assess long‐term outcomes, only patients followed up for at least 12 months after treatment initiation were selected. Information on (i) demographics, (ii) CIDP subtype, (iii) mode of onset, (iv) comorbidities (rheumatological, orthopedic, cardiological, respiratory, or neurological) causing functional disability, (v) presence of diabetes or (vi) monoclonal gammopathy, (vii) treatments administered, and (viii) pre‐treatment disease duration (the time from first symptom onset to the first treatment) was collected. For the UK cohort, pre‐ and post‐treatment disability was assessed using the Overall Neuropathy Limitation Scale (ONLS, 0–12), while the Korean cohort used the Inflammatory Neuropathy Cause and Treatment (INCAT, 0–10) score. As measures of muscle strength impairment, pre‐ and post‐treatment Medical Research Council sum score (MRCSS, 0–80) and post‐treatment Jamar grip strength (kg) were available for a subset of the UK and the Korean cohorts, respectively. Post‐treatment Inflammatory Rasch‐built Overall Disability Scale (I‐RODS, 0–100) was available for most subjects in both cohorts, allowing I‐RODS to be used as a unified outcome measure to analyze prognostic factors across cohorts.

### Statistical analysis

Continuous variables were presented as mean ± standard deviation or median (IQR), while categorical variables were presented as counts (%). Differences in clinical characteristics according to cohorts and the impact of treatment timing on outcomes were assessed using Student's *t*‐test or Mann–Whitney test (continuous variables), chi‐squared test, or Fisher's exact test (categorical variables), as appropriate. Correlation between variables was assessed using the Pearson correlation coefficient. To identify factors that independently influenced the outcome, a multiple linear regression analysis was performed, including variables with a *P*‐value <0.05 from the univariable linear regression. A subgroup analysis was performed to identify clinical characteristics specifically associated with early treatment benefit. A two‐tailed *P*‐value of less than 0.05 was considered significant. All statistical analyses were performed using R4.2.1.

### Approvals

This study was approved by the Institutional Review Board of Seoul National University Hospital and Boramae Medical Center (IRB No.: 1704–009‐842) and University Hospitals Birmingham (CARMS‐20702, October 23, 2023). Informed consent was waived due to the retrospective nature of the study.

## RESULTS

### Baseline characteristics

A total of 144 patients with CIDP were included (Korea: 74, UK: 70). Baseline characteristics such as age, sex, subtype distribution, duration of follow‐up, and comorbidities were similar to those previously reported (Table 1). Main differences between cohorts were the absence of distal CIDP in the UK cohort (*p* = 0.007), a longer delay to the first treatment in the Korean cohort (*p* = 0.04), and predominant use of IVIG in the UK and of corticosteroids in Korea as first‐line treatment (*p* < 0.001). Although the UK cohort showed a trend toward better outcomes as measured through the I‐RODS at follow‐up, this did not reach statistical significance (*p* = 0.051).

**TABLE 1:** Baseline characteristics of patients included in this study.

Characteristics	Overall (*n* = 144)	Korea (*n* = 74)	UK (*n* = 70)	*p*‐value
Age, mean (SD)	61 (14)	60 (14)	62 (14)	0.55
Female (*n*, %)	54 (37.5%)	28 (37.8%)	26 (37.1%)	0.93
Phenotype (*n*, %)				
Typical	111 (77.1%)	56 (75.7%)	55 (78.6%)	0.68
Multifocal	16 (11.1%)	6 (8.1%)	10 (14.3%)	0.24
Distal	8 (5.6%)	8 (10.8%)	0 (0%)	0.007*
Sensory‐predominant	6 (4.2%)	3 (4.1%)	3 (4.3%)	1.00
Motor‐predominant	3 (2.1%)	1 (1.4%)	2 (2.9%)	0.61
Acute‐onset CIDP (*n*, %)	38 (26.4%)	23 (31.1%)	15 (21.4%)	0.19
Comorbidity (*n*, %)	39 (27.1%)	21 (28.4%)	18 (25.7%)	0.72
Diabetes at diagnosis (*n*, %)	35 (24.3%)	18 (24.3%)	17 (24.3%)	1.00
Monoclonal gammopathy at diagnosis (*n*, %)	17 (11.8%)	11 (14.9%)	6 (8.6%)	0.24
Time to treatment (months), median (IQR)	9 (4–36)	12 (5–52)	7 (3–18)	0.04*
Follow‐up duration (years), median (IQR)	6.0 (3.6–8.6)	6.8 (3.1–11.4)	5.7 (3.7–7.8)	0.15
First‐line treatment (*n*, %)				
IVIG	70 (48.6%)	22 (29.7%)	48 (68.6%)	<0.001*
Corticosteroids	70 (48.6%)	51 (68.9%)	19 (27.1%)	<0.001*
IVIG + Corticosteroids	1 (0.7%)	1 (1.4%)	0 (0%)	1.00
Plasma exchange	3 (2.1%)	0 (0%)	3 (4.3%)	0.11
Active treatment at follow‐up (*n*, %)	77 (53.5%)	41 (55.4%)	36 (51.4%)	0.23
I‐RODS at follow‐up^a^	61 (46–88)	57 (42–83)	64 (48–100)	0.051
ONLS (0–12), median (IQR)				NA
Baseline			5 (4–7)	
Follow‐up			2 (0–3)	
INCAT (0–10), median (IQR)				NA
Baseline		3 (2–5)		
Follow‐up		2 (1–4)		
MRCSS (0–80), median (IQR)^b^				NA
Baseline			64 (57–70)	
Follow‐up			80 (73–80)	
Grip strength at follow‐up (kg), mean (SD)^c^	24.1 (11.0)	23.4 (10.8)	25.5 (11.5)	0.60

*Note*: Statistically significant values are expressed as asterisks (*).

^a^ 50 Korean and 56 UK subjects.

^b^ 63 UK subjects.

^c^ 48 Korean and 22 UK subjects.

Abbreviations: CIDP, chronic inflammatory demyelinating polyradiculoneuropathy; IQR, interquartile range; I‐RODS, Inflammatory Rasch‐built Overall Disability Scale; MRCSS, Medical Research Council sum score; ONLS, Overall Neuropathy Limitation Scale; INCAT, Inflammatory Neuropathy Cause and Treatment; SD, standard deviation.

### Impact of treatment timing on post‐treatment disability and response amplitudes

**FIGURE 1:**
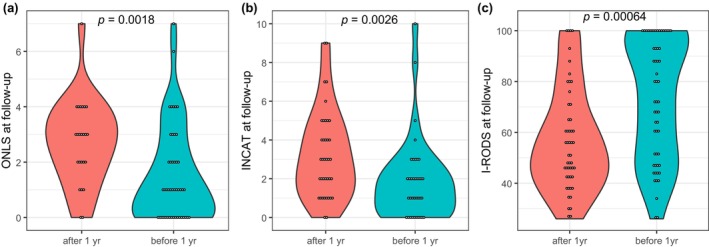
Impact of treatment timing on outcomes in patients with CIDP: (a) ONLS at follow‐up (UK, *n* = 70), (b) INCAT at follow‐up (Korea, *n* = 74), and (c) I‐RODS at follow‐up (UK, *n* = 56; Korea, *n* = 50). ONLS, Overall Neuropathy Limitation Scale; INCAT, Inflammatory Neuropathy Cause and Treatment; I‐RODS, Inflammatory Rasch‐built Overall Disability Scale.

UK patients who received their first treatment within 1 year of onset showed better ONLS at follow‐up than those who were treated after 1 year (*p* = 0.0018) (Figure 1a). Similarly, in Korean patients, treatment within 1 year was associated with a better INCAT score at follow‐up (*p* = 0.0026) (Figure 1b). Analysis of 106 patients of the combined cohorts with I‐RODS at follow‐up also showed consistent results (*p* = 0.00064) (Figure 1c). The amplitude of treatment response, assessed by the degree of improvement in ONLS and INCAT scores, was greater when treatment was commenced within 1 year (*p* = 4.3 × 10^−5^ and *p* = 0.012), in patients from the UK and Korea, respectively (Supplementary Figure 2).

### Impact of treatment timing on response rates

Considering the published minimum clinically important difference (MCID) cut‐offs for INCAT and ONLS scales (1 point for both), [14, 15] there were no significant differences between patients having received treatment within, or after 1 year, in Korean (*p* = 0.079), or UK (*p* = 0.081) cohorts. However, considering an improvement of ≥2 points on both scales to define responder status, significantly more patients were classified as responders if treated within 1 year versus after 1 year, in both Korean (*p* = 0.005) and UK (*p* < 0.001) cohorts.

### Impact of treatment timing on muscle strength impairment

In addition to disability, the impact of treatment timing showed a similar trend in muscle strength impairment (Supplementary Figure 3). Subjects treated within 1 year of onset had significantly better grip strength at follow‐up compared with those treated later (48 Korean and 22 UK subjects, *p* = 0.014) and showed greater improvement in MRCSS from baseline (63 UK subjects, *p* = 0.019). Although the association between MRCSS at follow‐up and treatment timing did not reach statistical significance (63 UK subject, *p* = 0.12), a clear difference was observed when analyzing only 49 subjects with typical CIDP (*p* = 0.0092).

### Inter‐correlations between clinical factors

Baseline factors and outcomes were intricately interrelated (Supplementary Figure 4). Time to treatment was associated with age (*r* = 0.34), mode of onset (*r* = −0.27), and I‐RODS at follow‐up (*r* = −0.34). Additionally, age and comorbidity, two variables related to I‐RODS outcomes, were correlated with each other (*r* = 0.23). The use of IVIG as first‐line therapy was associated with higher I‐RODS at follow‐up (*r* = 0.22) and a higher likelihood of treatment withdrawal (*r* = −0.27). IVIG use was influenced by the presence of diabetes (*r* = 0.40) and absence of comorbidity (*r* = −0.19).

### Univariate and multivariate linear regression for prognostic factors

Univariate and multivariate linear regression analyses are shown in Table 2. In the univariate regression, shorter time to treatment (*p* < 0.001), younger age (*p* = 0.003), absence of comorbidities (*p* = 0.004), and use of IVIG as first‐line therapy (*p* = 0.024) were associated with better prognosis. Among these, only time to treatment (*p* = 0.004) and presence of comorbidities (*p* = 0.020) remained significant in the multiple regression analysis.

**TABLE 2:** Factors associated with I‐RODS at follow‐up in 106 subjects (merged cohort).

Variable	Univariate		Multivariate	
	Estimate	*p*‐value	Estimate	*p*‐value
Time to treatment (months)	−0.159	<0.001*	−0.128	0.004*
Age (year)	−0.505	0.003*	−0.238	0.170
Phenotype (typical)	−4.690	0.410		
Acuteness	5.391	0.303		
Comorbidity	−14.556	0.004*	−11.266	0.020*
Diabetes at diagnosis	−3.436	0.517		
Monoclonal gammopathy at diagnosis	−4.424	0.560		
IVIG as the first treatment	10.375	0.024*	6.553	0.127
Follow‐up duration (month)	−0.002	0.963		
Ethnicity (Caucasian vs. Asian)	8.978	0.051		

*Note*: Statistically significant values are presented in asterisks (*).

Abbreviation: IVIG, intravenous immunoglobulin.

Linear regression analyses accounting for pre‐treatment disability (ONLS for the UK and INCAT for Korea) demonstrated that time to treatment was a consistent independent prognostic factor in both cohorts individually, together with pre‐treatment disability level and the presence of comorbidities (Supplementary Tables 1 and 2).

### Clinical characteristics associated with early treatment benefit

In subgroup analyses (Table 3), association between early treatment initiation and better I‐RODS outcomes was significant in patients aged 60 years or older (*p* = 0.002), but not in those who were younger (*p* = 0.26), in subjects with typical CIDP (*p* < 0.001), but not those with variant forms (*p* = 0.138), and in subjects with a chronic mode of onset (*p* < 0.001), but not those with acute‐onset disease (*p* = 0.233). Of note, the benefit of early treatment was consistent regardless of the pre‐treatment disability level as assessed by ONLS (UK) or INCAT (Korea), or the type of first‐line treatment agent used.

**TABLE 3:** Subgroup analysis to identify patient characteristics that benefit from early treatment initiation.

Variable	Coefficient	*p*‐value	95% CI
Age			
Age <60 (*n* = 59)	−0.157	0.260	−0.426 to 0.112
Age ≥60 (*n* = 85)	−0.132	0.002*	−0.214 to −0.050
Phenotype			
Typical (*n* = 111)	−0.193	<0.001*	−0.290 to −0.096
Variant (*n* = 33)	−0.109	0.138	−0.247 to 0.029
Mode of onset			
Acute (*n* = 38)	−0.187	0.233	−0.487 to 0.113
Chronic (*n* = 106)	−0.160	<0.001*	−0.246 to −0.074
Initial treatment			
IVIG (*n* = 71)	−0.174	0.004*	−0.287 to −0.060
Corticosteroids (*n* = 73)	−0.117	0.044*	−0.229 to −0.006
Pre‐treatment disability			
INCAT or ONLS ≥4 (*n* = 88)	−0.191	0.004*	−0.317 to −0.065
INCAT or ONLS <4 (*n* = 56)	−0.153	0.004*	−0.250 to 0.055

*Note*: Statistically significant values are expressed as asterisks (*).

Abbreviations: CI, confidence interval; IVIG, intravenous immunoglobulin; INCAT, Inflammatory Neuropathy Cause and Treatment; ONLS, Overall Neuropathy Limitation Scale.

## DISCUSSION

In this multicenter cohort study of 144 CIDP patients, we found that initiation of the first treatment within 1 year from onset was associated with milder post‐treatment disability as well as greater amplitude of treatment responses. The prognostic value of treatment timing remained significant even after adjusting for potential confounding variables. Additional predictors of CIDP outcomes observed were presence of functionally disabling comorbidity and pre‐treatment disability levels.

Our study provides insights into optimizing routine care to improve outcomes in CIDP. A timely intervention has been emphasized in various neuroimmunological disorders. [16, 17, 18, 19] From a pathological perspective, in CIDP patients with active disease, once macrophages strip the myelin sheath, risk of secondary axonal degeneration follows. [20, 21] Axonal damage can have a greater impact on disability than demyelination itself. [22] Early treatment, in this context, may help control neural inflammation before irreversible axonal damage accumulates. Additionally, it may halt the propagation of autoimmune responses by preventing epitope spreading [23, 24].

Our subgroup analyses provide additional valuable insights into which patients are most likely to benefit from early treatment initiation. The effect was particularly evident in older subjects, possibly due to reduced capacity for axonal regeneration and reinnervation, or a lower density of myelinated fibers at baseline. [25, 26] The lack of a significant impact of treatment timing in CIDP variants could be due to heterogenous pathophysiology among subtypes, or a type 2 error resulting from the smaller sample size in this group. [27, 28, 29] With regard to acuteness of onset, it is possible that treatment timing did not appear to impact on outcome, as treatment is usually commenced rapidly in such cases, compared with in cases of chronic onset. Importantly, the benefit of early treatment was consistent regardless of pre‐treatment disability levels or the type of first‐line therapy, highlighting the appropriateness of early treatment initiation of any treatment to achieve better outcomes, even in patients with milder symptoms, especially those with typical CIDP presenting insidiously.

Aside from treatment timing, other predictors of outcomes included comorbidity, pre‐treatment disability, age, and the use of IVIG as first‐line therapy. Of these, the latter two did not retain significance in multivariate regression analysis. A possible explanation is that older age increases the likelihood of comorbidities, resulting in a confounding effect. Regarding the type of first‐line therapy, the lack of its significance in multivariate analysis may align with current evidence, where none of the three standard options have established superiority. [13] Of note, in our combined cohort, use of IVIG as the first treatment was associated with a higher probability of treatment discontinuation. This contradicts findings from previous observations that found IVIG use to be associated with treatment dependence. [11, 30, 31] However, due to the retrospective nature of our analysis, where treatment decisions such as maintenance vs. switching were influenced by initial treatment responses, it is difficult to draw definitive conclusions.

In our cohort, nearly half of patients were off therapy after a median follow‐up of 6 years, which is relatively higher than previously reported remission rates. The PREDICT trial and its extension study observed remission or cure in 40% and one‐third of patients, respectively. [30, 32] Similarly, the international CIDP outcome study reported that 36% of patients achieved remission by 1 year. [33] On the contrary, other studies have reported higher remission rates (40 ~ 61%), indicating that the likelihood of remission can vary depending on treatment protocols, follow‐up duration, target population (whole vs. treatment naïve vs. treatment responders), and outcome measures used to determine remission. [6, 11, 34] It is also important to note that, in real‐world practice, treatment discontinuation can occur for reasons other than remission, such as treatment ineffectiveness, intolerance, or patient refusal [35].

There were some clinical differences between the UK and Korean patients. The most notable one was the type of first‐line treatment used. The UK cohort predominantly started with IVIG, whereas corticosteroids were most frequently used in Korea. This is likely due to the Korean insurance policy, which does not cover IVIG as a first‐line treatment option. The longer delay to the first treatment in Korea could be attributed to delays in patients accessing neuromuscular specialists or a lack of awareness of the clinical and electrophysiological features of CIDP. The absence of distal CIDP in the UK cohort could be due to ethnic differences, or a perceived relative lack of need for treatment in distal CIDP, in UK practice.

There are several limitations to our study. First, the number of participants was insufficient to analyze CIDP subtypes separately. The retrospective design led to the use of different outcome measures across cohorts, although I‐RODS was available at follow‐up for most subjects in both cohorts and provided confirmatory results. The treatment regimen, such as maintenance dose and frequency, treatment duration, and withdrawal strategies, was individualized and varied across patients, and therefore, the impact of these factors on outcomes could not be ascertained. However, we conducted a detailed analysis using multiple outcome measures validated for CIDP, whereas previous studies primarily relied on the mRS. [6, 8, 9] Furthermore, multivariate analyses allowed us to better understand the independent effect of each variable, and the subgroup analyses provided some insight into which patients are most likely to benefit from early intervention.

In conclusion, our findings highlight the importance of early treatment initiation in CIDP to achieve lesser post‐treatment disability and greater treatment responses. Time to treatment, disabling comorbidities, and pre‐treatment disability were identified as key prognostic factors. Further prospective studies with larger cohorts are needed to refine our understanding and optimize patient management.

## AUTHOR CONTRIBUTIONS

**Young Gi Min:** Conceptualization; methodology; data curation; investigation; validation; formal analysis; visualization; project administration; writing – review and editing; writing – original draft. **Jaehyun Jeon:** Investigation; data curation; supervision. **Sung‐Min Kim:** Conceptualization; data curation; supervision. **Yoon‐Ho Hong:** Investigation; data curation; supervision. **Christina Englezou:** Investigation; data curation; supervision. **Jung‐Joon Sung:** Conceptualization; funding acquisition; investigation; supervision; project administration. **Yusuf A. Rajabally:** Conceptualization; methodology; data curation; investigation; writing – original draft; writing – review and editing; validation; formal analysis; supervision; project administration.

## FUNDING INFORMATION

This work was supported by the National Research Foundation of Korea (NRF) Grant funded by the Korean Government (grant number: 2018R1A5A2025964). The UK investigators received no funding for this work.

## CONFLICT OF INTEREST STATEMENT

Y.A.R. has received consultancy honoraria from Sanofi, Janssen, Argenx, LFB, Polyneuron, Grifols, Takeda, and Dianthus, has received educational sponsorships from LFB and CSL Behring, and has obtained research grants from LFB. The other authors have no disclosures.

## Supporting information


Data S1.

## Data Availability

The data sets generated during and/or analyzed during the current study are available from the corresponding author upon reasonable request.
