# Supplementary material for: Determinants of long‐term disability in chronic inflammatory demyelinating polyradiculoneuropathy: A multicenter Korea/UK study of 144 patients
Source: Eur J Neurol. 2024 Dec 9;32(1):e16575. doi: 10.1111/ene.16575 (PMC11628630; doi:10.1111/ene.16575)
Supplement: Supplementary file 1 — Data S1. [file ENE-32-e16575-s001.docx]

**Supplementary Figure 1. Flow diagram of the present study**

**
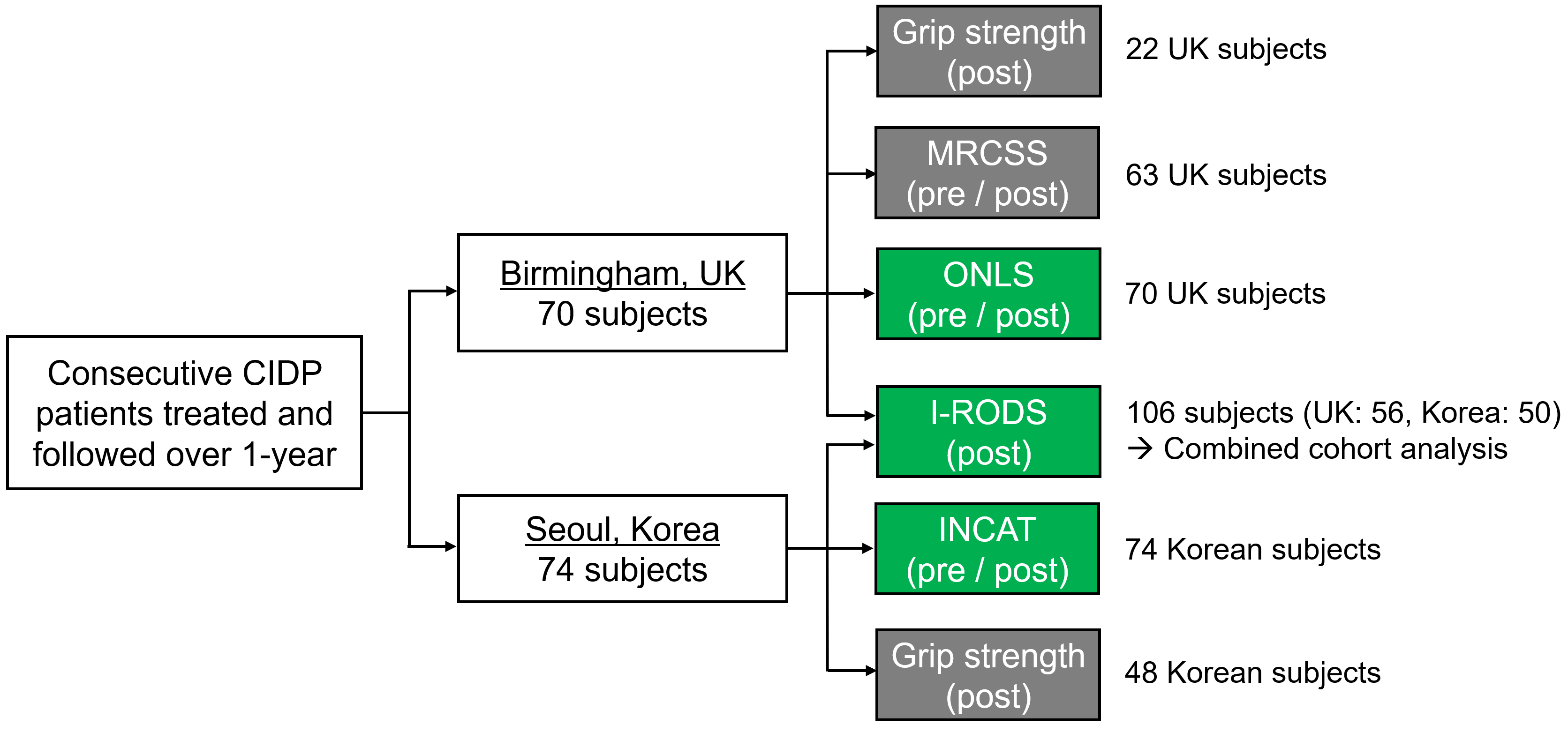
**

*Abbreviations: CIDP, chronic inflammatory demyelinating polyradiculoneuropathy; MRCSS, Medical Research Council sum score; ONLS, Overall Neuropathy Limitation Scale; I-RODS, Inflammatory Rasch-built Overall Disability Scale; INCAT, Inflammatory Neuropathy Cause and Treatment.*

**Supplementary Figure 2. Impact of treatment timing on the amplitude of treatment responses: (A) ONLS (UK, 70 subjects), (B) INCAT improvement from baseline (Korea, 74 subjects).**


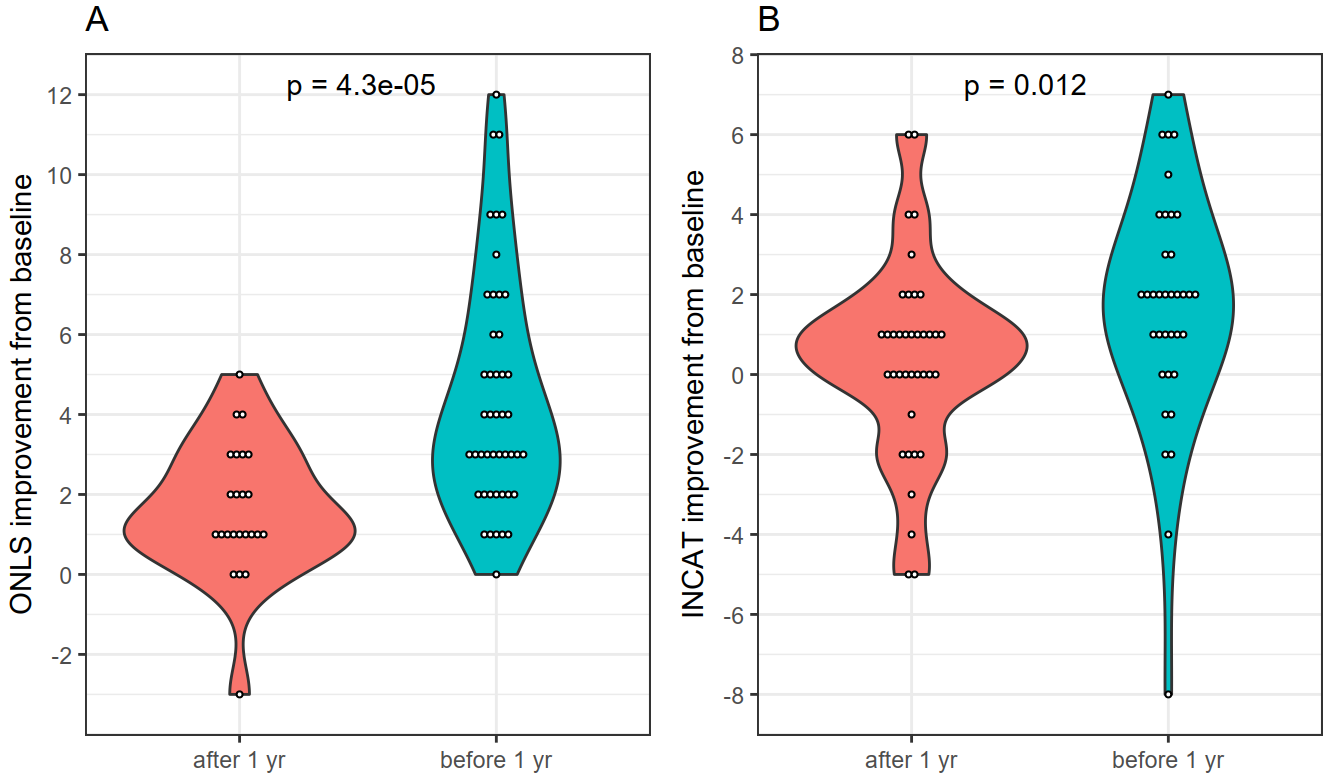


*Abbreviations: ONLS, Overall Neuropathy Limitation Scale; INCAT, Inflammatory Neuropathy Cause and Treatment.*

**Supplementary Figure 3. Impact of treatment timing on muscle strength impairment: (A) grip strength (kg) at follow-up (48 Korean and 22 UK subjects), (B) MRCSS at follow-up and (C) MRCSS improvement from baseline (63 UK subjects).**

**
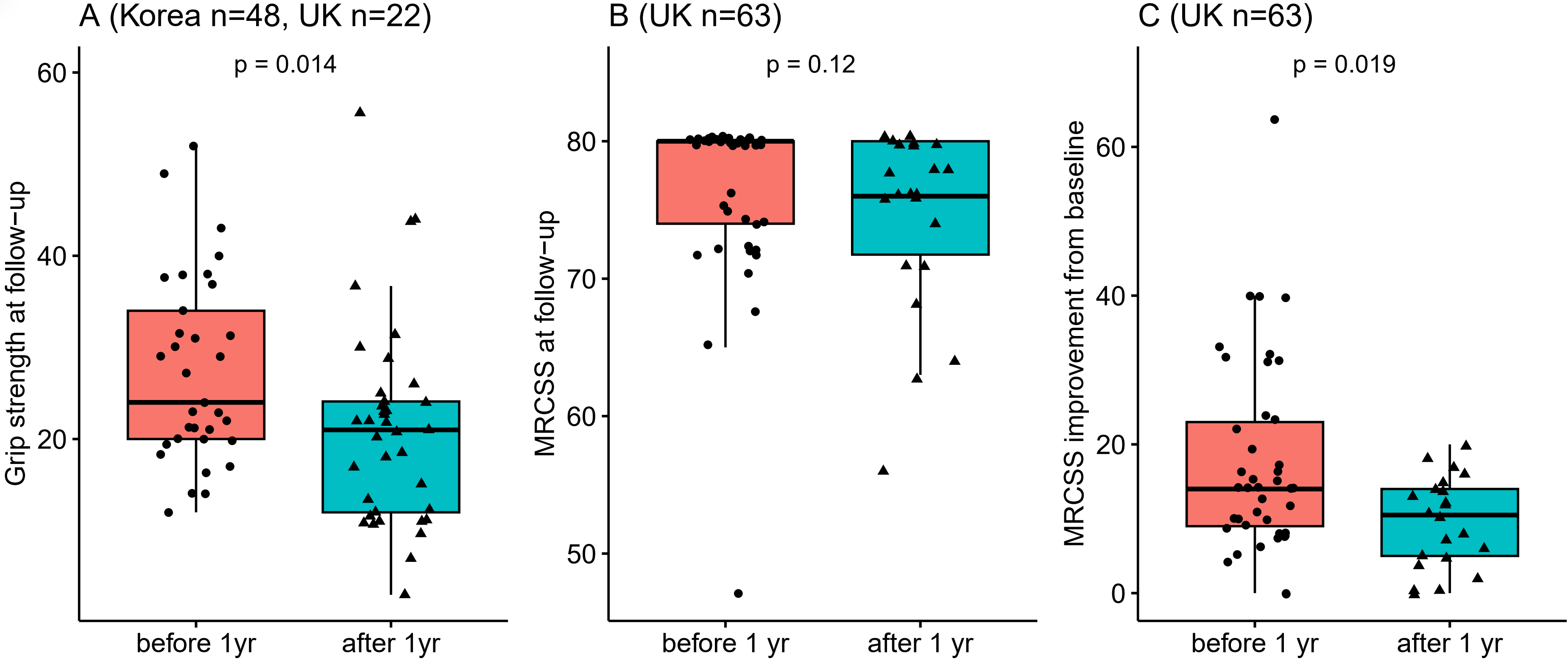
**

*Abbreviations: MRCSS, Medical Research Council sum score.*

**Supplementary Figure 4. Pairwise correlation matrix of baseline clinical factors and outcomes. The numbers and colors within the box represent Pearson correlation coefficients, with non-significant (p>0.05) pairs displayed as blanks.**

**
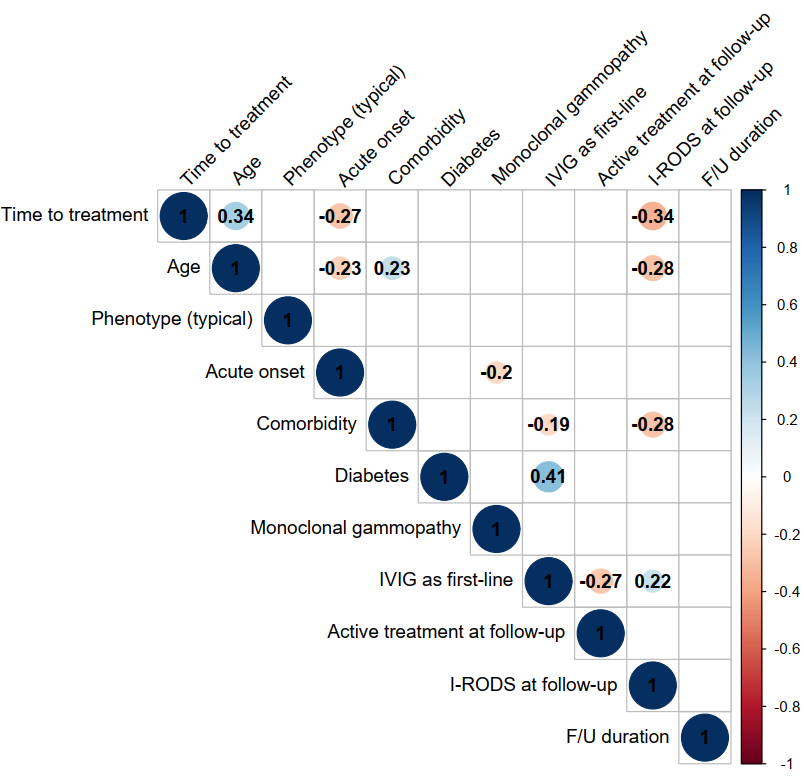
**

*Abbreviations: IVIG, intravenous immunoglobulin; I-RODS, Inflammatory Rasch-built Overall Disability Scale; F/U, follow-up.*

**Supplementary Table 1. Factors associated with ONLS outcomes (UK, 70 subjects).**

| **Variable** | **Univariate** | | **Multivariate** | |
| --- | --- | --- | --- | --- |
|  | **Estimate** | **P-value** | **Estimate** | **P-value** |
| **ONLS at follow-up** | | | | |
| Time to treatment (months) | 0.012 | 0.030^*^ | 0.011 | 0.050^*^ |
| Age (year) | 0.004 | 0.783 |  |  |
| Phenotype (typical) | -0.194 | 0.707 |  |  |
| Acuteness | -0.654 | 0.202 |  |  |
| Comorbidity | 0.115 | 0.812 |  |  |
| ONLS at diagnosis | 0.077 | 0.369 |  |  |
| Diabetes at diagnosis | 0.346 | 0.482 |  |  |
| Monoclonal gammopathy at diagnosis | 0.458 | 0.544 |  |  |
| IVIG as first treatment | -0.921 | 0.040^*^ | -0.808 | 0.067 |
| Follow-up duration (months) | -0.002 | 0.749 |  |  |
| **ONLS improvement** | | | | |
| Time to treatment (months) | -0.026 | 0.006^*^ | -0.013 | 0.035^*^ |
| Age (year) | -0.056 | 0.019^*^ | -0.008 | 0.607 |
| Phenotype (typical) | 1.600 | 0.057 |  |  |
| Acuteness | 2.982 | <0.001^*^ | 0.270 | 0.649 |
| Comorbidity | -0.690 | 0.388 |  |  |
| ONLS at diagnosis | 0.919 | <0.001^*^ | 0.825 | <0.001^*^ |
| Diabetes at diagnosis | 0.251 | 0.759 |  |  |
| Monoclonal gammopathy at diagnosis | 0.229 | 0.855 |  |  |
| IVIG as first treatment | 1.926 | 0.009^*^ | 0.793 | 0.098 |
| Follow-up duration (months) | -0.023 | 0.060 |  |  |

*Abbreviations: IVIG, intravenous immunoglobulin.*

**Supplementary Table 2. Factors associated with INCAT outcomes (Korea, 74 subjects).**

| **Variable** | **Univariate** | | **Multivariate** | |
| --- | --- | --- | --- | --- |
|  | **Estimate** | **P-value** | **Estimate** | **P-value** |
| **INCAT at follow-up** | | | | |
| Time to treatment (months) | 0.012 | 0.017^*^ | 0.010 | 0.029^*^ |
| Age (year) | 0.035 | 0.071 |  |  |
| Phenotype (typical) | 1.062 | 0.091 |  |  |
| Acuteness | -0.338 | 0.565 |  |  |
| Comorbidity | 1.848 | 0.002^*^ | 1.713 | 0.003^*^ |
| INCAT at diagnosis | 0.102 | 0.532 |  |  |
| Diabetes at diagnosis | -0.694 | 0.272 |  |  |
| Monoclonal gammopathy at diagnosis | 0.279 | 0.716 |  |  |
| IVIG as first treatment | -0.180 | 0.762 |  |  |
| Follow-up duration | -0.0002 | 0.967 |  |  |
| **INCAT improvement** | | | | |
| Time to treatment (months) | -0.015 | 0.014^*^ | -0.278 | 0.077 |
| Age (year) | -0.046 | 0.045^*^ | -0.013 | 0.521 |
| Phenotype (typical) | -0.038 | 0.960 |  |  |
| Acuteness | 1.664 | 0.015^*^ | 0.045 | 0.942 |
| Comorbidity | -2.564 | <0.001^*^ | -1.833 | 0.002^*^ |
| INCAT at diagnosis | 0.898 | <0.001^*^ | 0.761 | <0.001^*^ |
| Diabetes at diagnosis | 0.625 | 0.406 |  |  |
| Monoclonal gammopathy at diagnosis | -1.420 | 0.115 |  |  |
| IVIG as first treatment | -0.556 | 0.431 |  |  |
| Follow-up duration | -0.0003 | 0.938 |  |  |
